# Evolution of Electrospinning in Liver Tissue Engineering

**DOI:** 10.3390/biomimetics7040149

**Published:** 2022-09-30

**Authors:** Ashwini Vasudevan, Dinesh M. Tripathi, Subramanian Sundarrajan, Jayarama Reddy Venugopal, Seeram Ramakrishna, Savneet Kaur

**Affiliations:** 1 Department of Molecular and Cellular Medicine, Institute of Liver and Biliary Sciences, New Delhi 110070, India; 2 Department of Mechanical Engineering, National University of Singapore, Singapore 117581, Singapore; 3 Faculty of Industrial Sciences and Technology, Universiti Malaysia Pahang, Pekan 26600, Malaysia

**Keywords:** liver tissue engineering, electrospinning, nanofibers, natural and synthetic polymers, extracellular matrix proteins, hepatocytes

## Abstract

The major goal of liver tissue engineering is to reproduce the phenotype and functions of liver cells, especially primary hepatocytes ex vivo. Several strategies have been explored in the recent past for culturing the liver cells in the most apt environment using biological scaffolds supporting hepatocyte growth and differentiation. Nanofibrous scaffolds have been widely used in the field of tissue engineering for their increased surface-to-volume ratio and increased porosity, and their close resemblance with the native tissue extracellular matrix (ECM) environment. Electrospinning is one of the most preferred techniques to produce nanofiber scaffolds. In the current review, we have discussed the various technical aspects of electrospinning that have been employed for scaffold development for different types of liver cells. We have highlighted the use of synthetic and natural electrospun polymers along with liver ECM in the fabrication of these scaffolds. We have also described novel strategies that include modifications, such as galactosylation, matrix protein incorporation, etc., in the electrospun scaffolds that have evolved to support the long-term growth and viability of the primary hepatocytes.

## 1. Introduction

**Figure 1:**
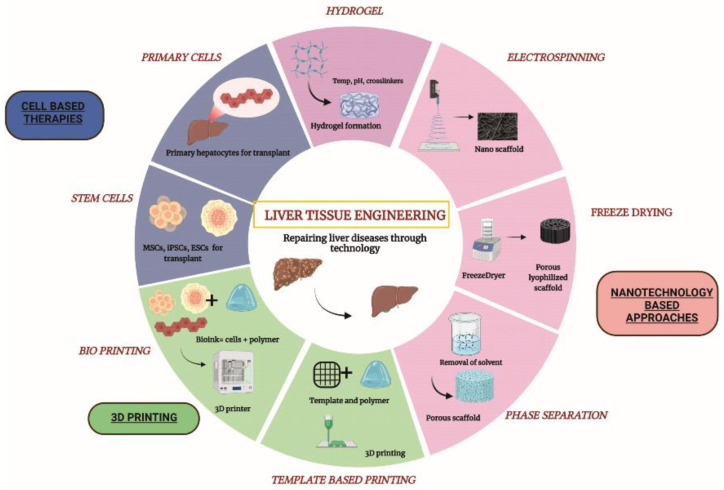
Different approaches used in liver tissue engineering. Three broad areas under liver tissue engineering categorized as cell-based approaches, 3D printing and nanotechnology-based approaches. MSCs—Mesenchymal Stem cells; iPSCs—induced pluripotent stem cells; ESCs—Embryonic Stem Cells. (Unpublished original picture by authors created using Biorender.com).

‘Nanotechnology’ came into existence in the year 1974 and today has advanced in almost all the fields of science, namely, medicine, metals, textiles, waste management, electronics and tissue engineering [1]. Nanotechnology deals with the study of particles less than 100 nm in diameter. Nanoparticles may vary in terms of shape and size and also properties such as optical activity, reactivity and toughness. Due to their high surface area and fine-tunable properties, nanoparticles have achieved widespread success in diverse scientific applications. Nanotechnology has revolutionized the field of tissue engineering by producing biomimetic nanofiber scaffolds. Nanofiber scaffolds are prepared with various types of materials, such as ceramics, metals, natural and synthetic polymers, to create nanofibers and nanopatterns. These scaffolds are now gaining popularity as their biological and topographical properties closely mimic the extracellular matrix (ECM) properties of the tissues [2,3]. Cell–matrix interactions are crucial for the optimal functioning of any tissue and nanofibrous scaffolds can provide this matrix substrate for the adhesion and proliferation of cells. Nanopolymers provide the most appropriate microenvironment for cell growth and are now being used for a plethora of tissue engineering applications including the creation of tissue implants for regenerative medicine, physiological tissue scaffolds for disease modeling, drug screening etc. [4,5]. Several techniques are available to develop nanofibers and nanopatterned structures including electrospinning, particulate leaching, lithography, self-assembly, phase separation and freeze drying (Figure 1).

Electrospinning is preferred over the other scaffold fabrication techniques because the pore size can be controlled in electrospun fibers which cannot be regulated in other techniques, namely, self-assembly and particulate leaching, which produce microporous structures. Electrospinning can fabricate user-defined scaffolds with optimum pore size as per the cell requirement. This technique also allows the development of scaffolds with naturally occurring polymers, such as alginate, gelatin and chitosan, as well as synthetic polymers. Nanofiber scaffolds produced from electrospinning have an orientation that mimics the dense collagen network of the natural ECM. Several studies have also proven that the orientation of the electrospun nanofibers promotes the attachment of cells by providing optimum spacing of integrin binding [6,7]. Electrospun nanofiber scaffolds have thus been widely used in the in vitro cultures. Additionally, unlike electrospinning, the rest of the techniques utilize high temperature and corrosive salts and chemicals, which may affect the biocompatibility of the fabricated scaffolds [8,9,10].

Nanofiber scaffolds produced by the electrospinning technique have been used in all fields of tissue engineering, to name a few, bone, cardiovascular, ligament and skin tissue engineering [11,12,13]. Aligned nanofibers produced by this technique are highly favorable for the growth of osteocytes. The mechanical stability of these nanofiber scaffolds also provides an added advantage during the in vivo transplantation of osteocytes [14,15,16,17]. The importance of electrospinning in the field of skin tissue engineering is its ability to fabricate the scaffolds as thin sheets that can be used as a patch to treat topical wounds. With this technique, we can also incorporate specific growth factors in the fabricated scaffolds and attempt adequate surface modifications for improved adhesion of the cultured cells [18].

The liver is the largest organ of the body and is involved in the metabolism of drugs and xenobiotics, detoxification, bile formation and energy synthesis functions. According to a recent study, liver diseases account for about 2 million deaths per year worldwide. Liver transplantation is the only option for patients with end-stage liver failure [19,20,21]. According to Health Resources and Service Administration (HRSA based in the USA), it has been reported that there has been a 10% increase in patients waiting for a liver transplant during the year 2021 (until September) globally. The list of waiting patients largely outnumbers the list of donor livers and only 1 out of 100 deserving liver disease patients finally receive a liver. The remaining mostly die with the want of a fully functional liver. Given the increasing burden of patients with end-stage liver diseases and the resulting dearth of suitable donor organs, scientists worldwide have been exploring other options, such as extracorporeal temporary liver assist devices [22,23,24], tissue engineering approaches, 3D printing and cell-based therapies, to combat liver diseases and most of these have also shown promising results in pre-clinical studies. The fabrication of scaffolds, implantable devices, cell encapsulated hydrogels and 3D printed liver tissues are being investigated under the umbrella of liver tissue engineering [25,26].

In the current review, we review technical aspects of electrospinning, its current use in liver tissue engineering and also its future potential in the field of liver tissue engineering.

## 2. Electrospinning

The invention of electrospinning dates back to 1980, by Yoshito Miura and group who were working on textile fibers [27], yet the use of electrospinning techniques in the field of tissue engineering to prepare nano-range scaffolds increased only in the past two decades [28]. A major advantage of this technique is that nanofibrous scaffolds with high surface area and porosity can be developed for the exchange of nutrients and oxygen and also allows infiltration of cells within the scaffold [29]. Electrospun fibers are in the nano range (from 100 nm to 50 µm) and have been observed to mimic the ECM architecture of a biological tissue. Several growth factors and drugs can be incorporated into these electrospun scaffolds through simple chemical modification of the surface [30,31,32,33,34,35,36], which can then be used as sustained drug releasing materials in vivo owing to their porous nature. Electrospun scaffolds are ideal for in vitro cultures and are also now being used for in vivo transplantation, especially in vascular reconstruction and skin tissue engineering [37,38,39]. Advancements in the field of electrospinning have given rise to its various subtypes, including coaxial electrospinning, multiple needle electrospinning, melt electrospinning, wet electrospinning and blend electrospinning. Core-sheath and hollow fibers can be produced by the coaxial spinning type, which has outer and inner spinnerets that can contain two different polymer solutions [40,41]. Blend electrospinning is different from coaxial spinning, where two polymers, or polymers with drugs or growth factors, can be blended and electrospun. Due to the toxicity of the nonpolar solvents used in a conventional electrospinning process, blending may result in the degradation of growth factors and active metabolites in drugs. To overcome this difficulty, two-phase electrospinning, where two different electrospinning methods are combined, is preferred, which allows the stability of growth factors and drugs to be maintained [42,43]. Melt electrospinning does not involve the use of toxic solvents, and, thus, is favorable for both in vitro cultures and in vivo conditions [44,45]. This process, however, requires very high temperatures to melt the polymer and only very few polymers are stable at high temperatures (e.g.: polycaprolactone and polyethylene). Wet electrospinning is another widely used electrospinning method which produces highly porous scaffolds with better cellular infiltration [46]. Conventional electrospinning systems have also been modified with multiple needles and multiple spinneret systems to fabricate scaffolds on a large scale.

**Figure 2:**
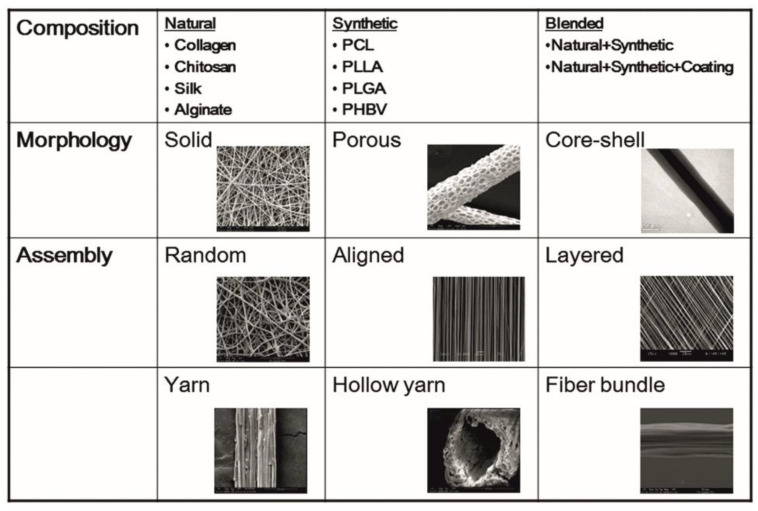
Patterns of the electrospun nanofibers categorized. Nanofibers can be electrospun in different formats such as random, aligned and layered depending on the instrumentation. Figure formatted with permission (NUSNNI, NUS).

A basic electrospinning set-up comprises of a syringe pump, syringe with blunt needle containing polymer solution, a collector and a high voltage current source. A high-intensity electric field (15 to 30 kV) is applied between two oppositely charged electrodes to set up electrospinning for scaffold production. One electrode is connected to the collector and the other is attached to the needle of the syringe containing the polymer solution. The flow rate at which the polymer solution is ejected out of the syringe pump is optimized according to the user’s experiment. The polymer is electrically charged as soon as it comes out of the nozzle as a spherical droplet. A charge–charge repulsion within the droplet creates a surface tension over the droplet, which is overcome by the high intensity electric field drawing the spherical droplet into a cone towards the collector [47] This is called Taylor cone formation, which is then followed by jet propagation. During jet propagation, solvent evaporation occurs and the charge within the jet increases with time and voltage. This causes instability of the jet and the fibers become patterned in the nanoscale range, which are then drawn towards the collector. The orientation of the patterned fibers formed depends on the collectors used. For example, the rotating drum collectors lead to the formation of aligned fibers, while static collectors would form random fibers [48] (Figure 2). In case of the wet electrospinning method, the fibers are collected in the water bath and rest of the set-up remains the same. Cell behavior varies drastically according to the surface topography of the electrospun scaffolds. Studies have reported that primary hepatocytes and cells of elongated phenotype-like myocytes and neuronal cells show improved cellular attachment and proliferation when cultured on aligned fibers, while non-elongated cell phenotypes are more proliferative on random fiber mats [49,50,51,52,53]. The seeded cells sense the surface changes through integrin receptor signaling (present on the surface of the cells) and different levels of receptor activation by different scaffolds cause a variability in cell adhesion and attachment.

Every component of the electrospinning setup can affect the formation of fibers and can also change the range/size at which the fibers are formed [54]. Rahmati et al. categorized various factors affecting fiber formation into three major groups, where the first group revolves around intrinsic properties of the materials used, including majorly the molecular weight of the polymer, viscosity and solvent nature. The molecular weight of the polymer plays a major role in determining the fiber formation and diameter. Increased molecular weight of the polymer increases the viscosity of the electrospinning solution. Highly viscous polymers tend to surpass the bending instability and form fibers with large diameters. The second group involves the processing parameters related to the equipment such as flow rate, distance between the needle and collector and voltage. Improper fiber formation may occur due to inadequate solvent evaporation when the flow rate and the distance are not adjusted. With increased flow rates and decreased distance between the collector and the needle, the solution in which the polymer is dissolved does not get the requisite time to evaporate, due to which a thick fiber mesh with inadequate pore size is formed. Jet propagation and solvent evaporation are thus two crucial factors that determine the fiber formation, which is governed by appropriate flow rates for developing several patterns of the fibers. The third category of factors affecting the electrospinning of fibers accounts for environmental factors such as humidity and temperature. Low temperature and high humidity in the air affect solvent evaporation and lead to improper fiber formation [55,56].

Polymers used for electrospinning of fibers can be natural (such as alginate, chitosan, silk, etc.) or synthetic PLA (polylactic acid), PLGA (polylactic co glycolide), PCL (polycaprolactone) or even both (hybrid polymers) [57,58]. The degradation products of PLA and PLGA polymers (lactic acid) are biocompatible. PCL is known for its non-toxicity and lower immunogenicity and cases where slower degradation of polymer is needed, such as in cases of nerve regeneration through tissue engineering [59]. The degradation products of PCL (caproic acid, succinic acid, valeric acid and butyric acid) have shown to be toxic for cell culture systems, but, surprisingly, PCL implants have been reported to perform well inside the host body [60]. The slow degradation rate of this polymer is the main reason for it to be used widely in drug delivery systems. PCL scaffolds are also porous, which allows improved growth of cells in the in vitro systems and also in vivo. When transplanted in vivo, endothelial cells can infiltrate and form new blood vessels to support angiogenesis and cell viability on PCL scaffolds. It has been reported that electrospinning of natural polymers (collagen, silk fibroin) alone results in a bead on string fiber formation and, hence, natural polymers are often mixed with a synthetic polymer to improve the mechanical properties of the formed fibers [61,62]. Overall, the high surface area of the electrospun scaffolds provide a suitable environment for cellular attachment and the nano size of the fibers that mimics the cellular protein size present in the natural tissue matrix improves the focal adhesion.

## 3. Hepatic Cell Types on Electrospun Nanofiber Scaffolds

Hepatocytes account for 80% of the hepatic volume and perform all the major functions of liver. The other cell types present in the liver are grouped as non-parenchymal cells (NPC), which mainly includes hepatic stellate cells, kupffer cells, sinusoidal endothelial cells and other cell types such as pit cells and cholangiocytes. NPCs hold for about 6.5% of the total hepatic volume. Sinusoidal endothelial cells are found on the lining of the space of Disse. They are different from the conventional endothelial cells due to the presence of fenestrae that facilitates the improved exchange of nutrients and oxygen. Kupffer cells are the major phagocytic cell type in the liver. Hepatic stellate cells are the reservoir of vitamin A and pit cells are the natural killer cells of the liver. Cholangiocytes are the cells lining the bile ducts [63]. The cellular architecture of the liver is supported by the extracellular matrix (ECM) with an array of several different macromolecules that together comprise the scaffolding of the liver. In a healthy liver, it forms only about 3% of its total area. The most abundant proteins of liver ECM are isotypes of collagen (I, III, IV and V), with different isotypes localized to different areas.

Prolonged cultures of viable and functional liver cells such as primary hepatocytes and hepatoma cell lines on nanofiber scaffolds is the major goal of liver tissue engineering. The survival of primary hepatocytes ex vivo has remained a challenge for years. Adult primary hepatocytes do not survive after 3–4 days of culture as they change their phenotype and transform into mesenchymal cell lineages. The use of electrospun scaffolds for liver tissue engineering was first reported in the 21st century [56,57,58,59,60,61]. Zhang-Qi Feng et al. were some of the few researchers who pioneered the culturing of liver cells on electrospun scaffolds [60,64,65]. Among the cell lines, HepG2 shows better efficiency when cultured on electrospun fibrous scaffolds, on both natural (silk fibroin) as well as (PCL) synthetic polymer. Other than the hepatoma cell lines, cryopreserved human primary hepatocytes and rat/mice isolated primary hepatocytes have also been cultured on electrospun scaffolds, however, with limited success. It has been observed that primary hepatocytes display better viability with the natural polymers or with synthetic polymer scaffolds when they are modified with matrix proteins such as collagen, fibronectin or RGD peptides [66,67,68,69,70,71]. Another bottleneck of culturing primary hepatocytes is their limited replicative potential in vitro. An effective approach that has now emerged is to grow these cells as spheroids. Bell et al. have shown that hepatocytes can be cultured as spheroids for up to 35 days without compromising their functionality [70]. Hepatocytes have also been seen to form spheroids when cultured on galactosylated surfaces [71,72,73,74,75,76,77]. Galactose–asialoglycoprotein receptor (ASGPR) present on the hepatocytes has been demonstrated to be a key player mediating this interaction. Kian-Ngiap Chua et al. used poly (e-caprolactone-co-ethyl ethylene phosphate) (PCLEEP) polymer for scaffold formation and surface-modified it with polyacrylic acid and -O-(60- aminohexyl)-D-galactopyranoside (AHG) for galactosylation [75]. Isolated rat hepatocytes began to form clusters after day 1 on these galactosylated scaffolds. This was not observed on the non-galactosylated substrate, where the cells took an irregular shape and topography. Functional analysis revealed that an increased secretion of albumin and urea synthesis was observed with hepatocytes cultured on galactosylated substrates after 2 days and P450 activity increased after day 5. This was a major advantage of the developed substrate as it has been reported that P450 activity of primary hepatocytes usually deteriorates with time in culture conditions otherwise [72,73]. Hong-Fang Lu et al. showed that hepatic spheroids co-cultured with non-parenchymal cells on galactosylated PVDF (Polyvinylidene fluoride) surface have enhanced P450 activity [74]. Several other studies also reported the efficiency of galactosylated surfaces in maintaining hepatic spheroid phenotype, function and preventing their trans-differentiation [75,76,77,78,79]. Another study by Kian-Ngiap Chua et al. employed a dual-functional scaffold for facilitating adhesion and enhanced functionality of the primary hepatocyte spheroids. 3-methylcholanthrene (3-Mc) is a selective inducer of P450, and this group prepared 3-Mc-loaded electrospun PCLEEP polymer scaffolds by mixing the inducer with the polymer solution before spinning them into scaffolds. The galactosylated surface helped in the prolonged culture of the hepatocytes as spheroids and the bio-molecule-loaded feature improved the functionality of hepatocytes. Galactosylated scaffolds showed 85% cell adhesion, whereas attachment was a little low with 3-Mc loaded scaffolds (76%) and very poor attachment was observed with the unmodified scaffolds (PCLEEP alone) (37%). The P450 function of the dual scaffolds increased by 1.5-fold in comparison with the galactosylated scaffolds, clearly demonstrating the usefulness of this approach [80]. Besides primary hepatocytes, bone marrow stem cells (BMSCs), human mesenchymal stem cells (hMSCs), etc., have also been used in liver tissue engineering, where these cells are seeded onto the electrospun fibrous scaffolds and are trans-differentiated into hepatocytes with appropriate growth factors. This approach has also extended the life span and functionality of the differentiated hepatocytes in vitro [81,82,83,84].

## 4. Electrospun Synthetic Polymers for Liver Cell Cultures

**Figure 3:**
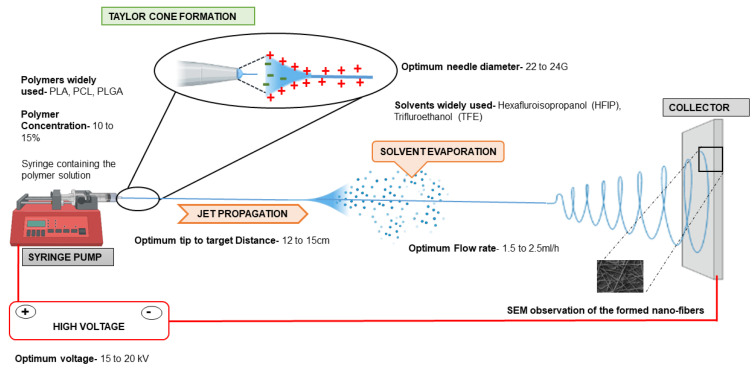
Optimum electrospinning parameters used for liver tissue engineering are provided. Polymers such as PLA, PCL and PLGA are most commonly used at a concentration of 10–15% dissolved in solvents such as HFIP, TFE, etc. The needle used for electrospinning has a diameter of 20–24 G and the tip to target distance should be about 12–15 cm. The voltage of the equipment should be maintained at 15–20 kV. The flow rate of the polymers should be 1.5 to 2.5 mL/h. With these parameters, the obtained fiber diameter and porosity is approximately around 400–450 nm and 40 µm, respectively, and is suitable for hepatic cells. PLA: Polylactic acid; PCL: Polycaprolactone; PLGA: Polylactic-co-glycolic acid; HFIP: Hexaflouro-isopropanol; TFE: Trifluroethanol (unpublished original pictures by authors created using Biorender.com)**.** PCL is another widely used polymer for liver tissue engineering applications.

Synthetic polymers used for liver tissue engineering are PLA, PLLA (Poly-L-Lactic Acid), PCL and PLGA. Figure 3 provides an overview of different polymers and technical electrospinning parameters used in liver tissue engineering. Among the synthetic polymers, PLA and PCL are the most preferred polymers. PLA was one of the first polymers used for tissue engineering applications. Liu and group have recently summarized applications of PLA in tissue engineering [82]. However, PLA alone does not effectively facilitate cellular attachment, thus, PLA is mostly combined with any natural polymer or matrix proteins and used for liver tissue engineering approaches. For example, a combination of PLA composite with silk fibroin has been reported for liver cell lines, HepG2 cells [83]. Liu et al. used lecithin- doped electrospun PLA scaffolds to achieve better cellular viability and functionality of HepG2 cells. Lecithin has been incorporated in this study since PLA lacks the biological component required for the proper attachment of cells. Increased cell viability and the highest cellular activity was observed in the group containing lecithin with PLA after 3 days of culture [67]. Collagen, being the most abundant protein in the matrix, has been widely incorporated with synthetic polymers to support hepatic cells. Hepatic differentiation of hBMSCs(human Bone Marrow derived stem cells) has been observed with scaffolds containing PLLA with collagen [85]. A study by Das et al. has shown that incorporating two majorly present ECM proteins, collagen and fibronectin, in different ratios with PLGA showed improved CYP gene activity of Huh7.5 hepatoma cells. They have employed wet electrospinning to obtain fibers with increased pore size in this study. The cells were cultured for 28 days on the modified scaffold and a tenfold increase in cell number was observed with the scaffold having the collagen to fibronectin protein ratio (C:F) of 3:1. Enhanced cell death was observed in the group without the ECM proteins (C:F = 0:0). CYP3A4 and CYP3A7 expression were 7.3-fold and 4.5-fold higher in the modified scaffold with the 3:1 ratio when compared to the unmodified scaffolds [46].

PCL is effective in facilitating cellular growth and proliferation. However, to overcome the hydrophobicity of this polymer and also to improve its mechanical properties, this polymer is also combined with natural polymers and/or tissue matrix proteins. PCL combined with collagen and PES polymer has shown effective hepatic differentiation of the hBMSCs [86]. Bishi et al. have shown that a co-polymer of these two polymers, PLA and PCL (PLACL), is most suitable for liver cells due to its highly lipophilic nature [87]. This study has used a blend of PLACL and collagen in the ratio (2:1) along with hMSCs for trans-differentiation into hepatocytes. The addition of collagen to the PLACL polymer resulted in a more hydrophilic polymer suitable for attachment of the hMSCs. The tensile strength was reduced after the collagen blend, yet the elastic modulus remained the same as that of the PLACL alone. The cell number was increased gradually on the PLACL/collagen scaffold starting from day 7 till day 28. The hMSCs’ phenotype was maintained till day 7 and, upon treatment with dexamethasone and other liver-specific growth factors, the cells attained polygonal morphology similar to the hepatocytes, which was maintained until day 28 on the PLACL/collagen scaffolds. The differentiation of the hMSCs into hepatocytes was also confirmed with an increased gene expression of hepatocyte nuclear factor 4 alpha (HNF4a) and albumin and a decreased expression of alpha-fetoprotein (AFP) from day 14 to 28. Albumin secretion was also enhanced in the hepatospheres grown on the PLACL/collagen scaffold at day 28. The extent of differentiation from hMSCs to hepatocytes was not very significant in scaffolds with PLACL alone. Hence, we can conclude that, although synthetic polymers are capable of supporting the culture of hepatic cells, the inclusion of a suitable biological component facilitates better attachment, adhesion and prolonged functional features of the cells on these scaffolds.

## 5. Electrospun Natural Polymers for Liver Cell Cultures

Natural polymers are also used in tissue engineering, but not as widely as synthetic ones, due to their compromised mechanical strength and problems in tunability according to the user’s experiment. Chitosan is one of the suitable natural polymers for hepatocytes, because it is structurally similar to the glycosaminoglycans present in the liver ECM. A wide variety of matrix proteins such as collagen, fibronectin, epibolin and chondronectins are used as coating for culturing hepatocytes in vitro, with collagen being the most widely used protein. However, collagen or fibronectin alone cannot be used as an electrospun scaffold, due to their poor mechanical strength. A study has reported the use fibronectin along with electrospun chitosan scaffolds [69]. Primary hepatocytes cultured on these fibronectin-coated chitosan films exhibited their characteristic polygonal morphology, whereas the cells cultured on 2D chitosan films alone maintained a round morphology. The same pattern was also observed between fibronectin-coated electrospun chitosan nanofibers and electrospun chitosan nanofibers alone. Cell viability confirmed with calcein-AM staining was markedly improved with fibronectin coating on electrospun chitosan nanofibers. Hepatocytes have a distinct feature of robust communication with their neighboring cells such as Kupffer cells, sinusoidal endothelial cells and fibroblasts. Their morphology and functions largely depend on their cross talk with these neighboring cells. In this study, the authors illustrated that the hepatocytes co-cultured with the fibroblasts on layers of chitosan fibers coated with fibronectin in a 3D environment maintained their morphology and enhanced albumin secretion for about 18 days in comparison to monoculture of the hepatocytes. The cell migration and adhesion were higher on the electrospun scaffolds than on the films, showing the efficiency of the porous nature of the nanofibrous scaffolds developed by the electrospinning technique. The P450 functional activity of the seeded cells was also increased with the 3D co-culture system of electrospun scaffolds, when compared to monoculture systems. P450 activity of hepatocytes is a crucial functional property of the cells to be considered, when the cells on scaffolds are meant to serve as a device for drug testing.

**Figure 4:**
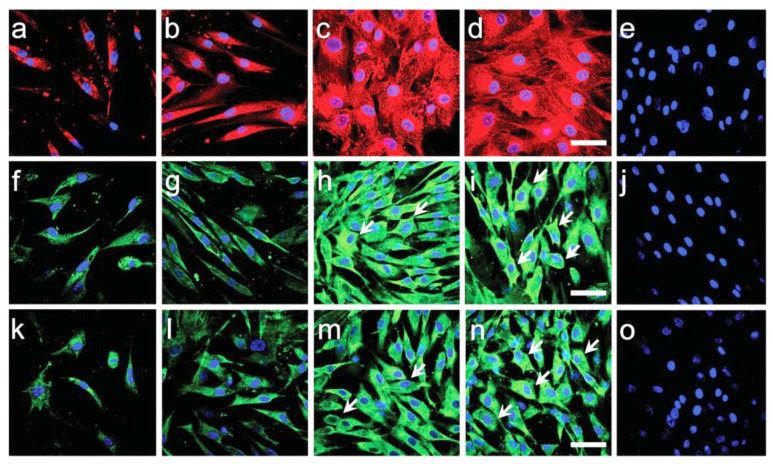
Reprinted with permission. Bishi DK et al., Adv Healthc Mater. 2016; 1058-70. Hepatocyte-specific marker expression (α-fetoprotein, albumin and cytokeratin-18) in BMSCs-derived hepatocyte-like cells as shown by confocal microscopy images (merged) on (**a**,**f**,**k**) PLLA scaffold with recombinant hepatic growth factor induction, (**b**,**g**,**l**) PLLA scaffold with hepatogenic serum induction, (**c**,**h**,**m**) PLLA/gelatin with recombinant hepatic growth factor induction, (**d**,**i**,**n**) PLLA/gelatin scaffold with hepatogenic serum induction at day 28. Alexa Fluor-594 labelled α-fetoprotein (**a**–**d**: red), Alexa Fluor-488 labelled albumin (**f**–**i**: green) and Alexa Fluor-488 labelled cytokeratin-18 (**k**–**n**: green) expression represents features of hepatocyte-like cells. (**e**,**j**,**o**) Undifferentiated BMSCs on PLLA/gelatin scaffolds did not express any hepatic markers. Nuclei were stained with DAPI (blue). Compared to PLLA scaffolds, more mature hepatocyte-like cells with cuboidal-to-polygonal morphology were observed (shown by white arrows) on PLLA/gelatin scaffolds in either induction condition (scale bar = 100 μm.) [81].

With evolving trends in electrospinning, natural polymers are now being blended with synthetic components to create scaffolds that can support cells in vitro as well as in vivo. Silk fibroin is one such biopolymer that has been widely explored by scientists for tissue engineering applications given its resemblance with collagen [66]. Electrospinning silk fibroin, however, often shows bead formation along with the nanofibers formed. To overcome this difficulty, PEO (Polyethylene Oxide) synthetic polymer has been incorporated along with silk fibroin and galactosylated chitosan for obtaining linear fibers without the beads. The advantage of using natural polymer is that, due to its hydrophilicity, it can retain water more than the synthetic scaffolds without much swelling [88]. Kasoju et al. have shown that the swelling degree of the silk-fibroin-based galactosylated polymer was only 2%, whereas its water retention capacity was 75%. HepG2 cells cultured on these modified silk-based scaffolds for a period of 7 days showed an increase in cell density with time. It was also observed that the cells attained a spheroid morphology with the help of galactosylated surface modifications on the scaffolds. Several other studies [79,80,81] have also reported that galactosylated surfaces can induce the spheroid formation of the cells on the natural polymers. Bishi et al. reported that human BMSCs successfully trans- differentiated toward functional hepatocytes on the nanofibrous scaffolds, which were formed by the combination of a natural polymer, gelatin and a synthetic polymer, PLLA [81]. The nanofibrous scaffolds supported cell adhesion, proliferation and efficient commitment of hBMSCs towards metabolically competent hepatocytes (with enhanced albumin secretion and CYP3A4 activity). The topography of PLLA/gelatin nanofibers guided cell morphogenesis through enhanced integrin attachment during hepatic differentiation of hBMSCs (Figure 4). This approach of using modified electrospun scaffolds thus holds great promise for creating disease-specific, ex vivo engineered liver tissues for clinical translation studies.

## 6. Liver Extracellular Matrix-Based Electrospun Scaffolds

Several studies are now focusing on incorporating the liver ECM as a whole or in part in the electrospun fibrous scaffolds for providing the growing hepatocytes with the native microenvironment. ECM is crucial for a cell to maintain its phenotype and any changes in its components can drastically change the morphology of a cell. In tumors, cells attaining migratory potential (transformation from epithelial to mesenchymal phenotype) and angiogenic properties are widely dependent on the changes in the ECM of tumor cells. Liver ECM is mainly composed of type I, II, IV and type IX collagen. Other than this, glycoproteins, such as laminin, fibronectin, tenascin and nidogen, and the normal proteoglycans heparin sulphate and chondroitin sulphate are also present in the ECM [89,90]. ECM-modified scaffolds can also be infused with hepatocyte-specific growth factors to achieve maximum functionality. Slivac et al. compared the viability of HepG2 cells when cultured on decellularized liver ECM scaffolds and on PCL mats. It was observed that the cell viability was greatly improved on the ECM scaffolds than on the PCL scaffolds [91] Grant et al. used decellularized human liver ECM and mixed it with the polymer solution to make blended electrospun scaffolds for THLE-3 liver cell lines. PLLA was used as the polymer in this study and the decellularized liver powder was mixed with 0.25 M acetic acid and then mixed with the polymer solution in the ratio of 1:9 and the electrospinning was performed at a 2.5 mL/h flow rate. The Young’s modulus of the scaffold revealed that the blended scaffold with the ECM was stiffer than the conventional polymer scaffold without the decellularized Liver ECM (dLEM). They observed better cell viability with the cells cultured on the dLEM scaffold in comparison with scaffold without dLEM at day 5. An increased albumin secretion by the hepatic cells cultured on the scaffold with dLEM was reported, while the scaffolds modified with the individual ECM components (collagen, fibronectin and laminin) did not show such improvements, indicating improved functionality of the hepatocytes when grown on whole liver matrix [92]. Earlier, this group also reported a novel method to produce dLEM-based scaffolds for cultures [93]. Here, electrospun PLA scaffolds were used, on which an initial layer of epithelial cells was cultured and transfected with human fibronectin vector. Later, the seeded epithelial layer of cells was decellularized with detergents such that the ECM components were intact on the PLA discs and then further HepG2 cells were grown on the decellularized PLA discs. Although the study put forward a novel approach of cell-derived ECM for hepatic cell cultures, the major limitation of this study is that remnant detergents on the scaffolds after the decellularization process might affect the viability of cultured hepatic cells and there is also a possibility that the scaffolds might be partially degraded if electrospinning had not been performed with care, and also if the concentration of the detergents was not optimized. Besides hepatic cell lines, researchers have also used primary hepatocytes on ECM-based electropun scaffolds. Most of the studies have shown to preserve hepatocytes in their original morphology for up to 7 days on these scaffolds. Brown et al. reported improved functions of primary human hepatocytes cultured on ECM-modified electrospun scaffolds, highlighting the role of matrix proteins in affecting primary hepatocyte morphology and function. This study used the wet electrospinning technique to obtain nanofibers with increased pore sizes. By this method, the fibers were collected in a liquid bath to avoid a dense accumulation of the fibers and increased pore size. The polymer used in this study was PLGA, which was surface-modified by EDC/NHS (N-(3-Dimethylaminopropyl)-N′- ethyl carbodiamide hydrochloride and N-hydroxysuccinimide) to allow increased attachment of the matrix proteins later attached on the scaffolds. The authors used both collagen and fibronectin in a ratio of 2:1 to mimic the composition of native liver matrix, which contains 60% collagen and 30% non-collagenous proteins. An average pore size of 30 µm was obtained by the wet electrospinning technique, which is the actual average pore size of a human liver tissue, while the pore size obtained by the conventional spinning technique was only 10 µm. Cross-sectional analysis of SEM revealed higher infiltration of the hepatocytes in the scaffolds prepared by wet electrospinning than those prepared by the conventional technique. Hepatocytes on the surface of scaffolds modified with matrix proteins showed better attachment, spreading and morphology when compared to the unmodified scaffolds. Expression of the albumin gene was 3.5-fold higher on collagen-modified scaffolds when compared to the unmodified scaffolds. The CYP3A4 activity of the hepatocytes cultured on the collagen-modified scaffolds was also increased by about fourfold. The albumin and urea secretion increased ten times from day 2 to day 14 on the collagen-modified scaffolds in comparison to the unmodified scaffolds. The study thus concluded that collagen-modified scaffolds are better than unmodified or fibronectin-modified scaffolds [94]. Bual et al. assessed the functions of primary rat hepatocytes cultured on PCL/gelatin electrospun scaffolds incorporated with decellularized porcine liver ECM. Tissue-like aggregation of the cultured hepatocytes was seen on the scaffolds with the highest amount of ECM incorporation at day 7. This was supported by increased albumin secretion and CYP enzyme activity on the same day [95]. Thus, incorporation of decellularized liver ECM blended with natural/synthetic polymers may serve as one of the most effective nanofiber scaffolds for maintaining the viability and functionality of primary hepatocytes. Table 1 summarizes different studies that has used electrospinning for liver tissue engineering.

## 7. Recent Innovative Approaches in Electrospinning for Liver Tissue Engineering

For prolonged cultures of hepatocytes, studies have now reported the use of hepatic cells in the form of 3D spheroids. Innovative approaches have been employed by researchers to incorporate the hepatocytes as spheroids, which is discussed in the following section. Wei et al. modified the conventional electrospinning method and came up with the idea of short fibers to support culture of hepatic spheroids without the need for surface modifications. They reported that the length of the fibers can be modified according to the length of the spheroids and showed that spheroids cultured on the PSMA (Poly(styrene-co-methyl acrylate) fibers of about 50µm length have improved drug metabolism and drug clearance [97]. Carbon nanotubes (CNTs) in the form of nanofibrous mats are known to provide electrically conductive surfaces and have been used for prolonged 3D spheroid cultures [98]. Koga et al. have reported that CNTs have the ability to induce the formation of hepatocyte spheroids [99]. Wei et al. have also used multiwalled CNTs functionalized with galactose moieties on the surface for efficient hepatic spheroid cultures. They demonstrated that hepatocytes cultured on these functionalized fibrous mats showed better functions, namely, better drug clearance and increased expression of drug metabolizing genes [100].

**Figure 5:**
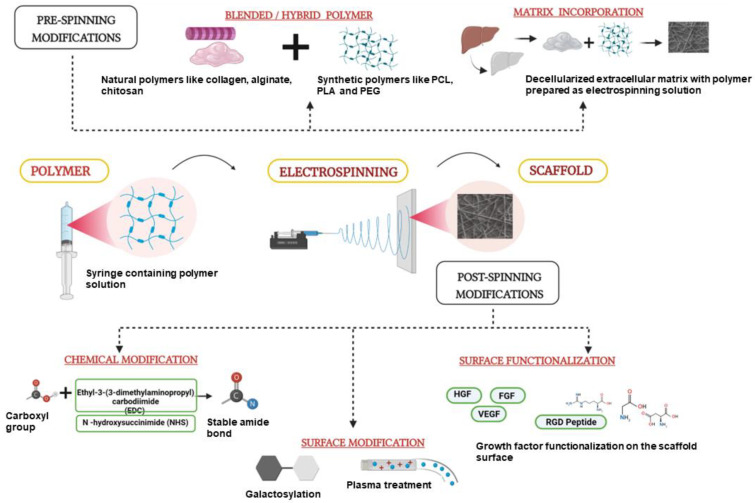
New Strategies employed in Electrospinning for Liver Tissue Engineering. Pre-spinning modifications including incorporation of matrix proteins, drugs or decellularized whole tissue matrix. Post-electrospinning strategies such as surface modification of the fibers with galactose, growth factors and RGD peptide conjugation, thereby improving the quality and functionality of the developed fibers for culture of liver cells. HGF—Hepatocyte Growth Factor, FGF—Fibroblast growth factor, VEGF—Vascular Endothelial Growth Factor (Unpublished original picture by authors created using Biorender.com).

Besides in vitro cultures, electrospun liver scaffolds are also being used for in vivo applications. The scaffolds can be fabricated as patches containing nano-fibrous mesh that can then be implanted at the site of injury. Kim et al. have recently shown that electrospun scaffold patches can be used to deliver healthy hepatic cells in toxin-induced liver injury mouse models. In this study, they used PCL for fabricating electrospun scaffolds/sheets and seeded them with patient-derived primary hepatocytes in a stacking manner by 3D bioprinting to mimic the native liver environment. The survival of the animals with the hepatic sheet transplant was 70% as compared to that of the control group without the scaffold. This study has opened the doors of using electrospun liver cell scaffolds for liver transplantation and in vivo therapy [96]. Another study by Salerno et al. has also proved the potential of electrospinning in mimicking the native liver tissue architecture with multiple cell types. They used the dry jet–wet electrospinning method to prepare hollow PCL fibers, and placed them in a bioreactor which contained an outer luminal segment where primary human hepatocytes were cultured and an inner luminal segment where endothelial cells were seeded in a hexagonal manner, thereby mimicking the native liver architecture [101]. The authors observed improved hepatic functions such as glucose consumption and albumin secretion for up to 18 days in the perfusion bioreactor. The recent studies on the electrospun scaffolds show the potential of them on the clinical front, an overview of the recent strategies employed for the fabrication of electrospun nanofiber scaffolds for liver cells is given schematically in Figure 5. Patient-specific scaffolds made out of this technique would give us the edge of replacing liver transplantation treatment with tissue engineering.

## 8. Conclusions

Reproducing the functions of the liver in total or in part for several downstream applications has remained a formidable task. The major hurdle in reproducing liver tissue ex vivo is the progressive loss of functions of the hepatocytes within a span of few days under these conditions. Electrospun fibrous scaffolds with a myriad of modifications serve as excellent cell-supporting substrates by providing nanoscale fibrous structures with interconnecting pores, resembling natural tissue ECM. Electrospinning with both natural and synthetic polymers and now also decellularized liver ECM has enormous potential in the development of liver tissue scaffolds with complex geometric/architectural structures. However, we still need many more advancements in the field. Culturing of hepatocytes on electrospun scaffolds as 3D spheroid cultures would lead to better growth and functions of hepatocytes as compared to 2D cultures. Additionally, a co-culture of hepatocytes along with other liver cells such as endothelial and hepatic stellate cells would impart better functionality to the hepatocytes. The success of the cell cultures on the fabricated scaffolds would also largely depend on the topology, composition and mechanical properties of the acellular scaffold. Properties such as optimum matrix stiffness and pore size would be important for achieving appropriate cell–ECM interactions. As our knowledge about the complexities of native liver ECM improves, newer modified biomaterials coupled with improved protocols for electrospinning would allow the fabrication of efficient scaffolds that would facilitate proliferation and differentiation of the primary hepatocytes and spheroids. Finally, the perfusion of the fabricated cell-seeded electrospun scaffolds under dynamic culture conditions in microfluidic devices would allow fabrication of the most apt working model of the liver sinusoid. Interdisciplinary collaborations would be necessary to undertake these challenges. The day is not far when these steps and developments in the field of electrospinning would allow us to reconstruct an efficient and functional liver tissue that could not only be used in vitro but also for clinical applications in vivo.

## Figures and Tables

**Table 1:** Summary of the different polymers and their electrospinning strategies used in liver tissue engineering to date.

Type of Polymer	Polymer	Cell Type	Modification	Electrospinning Method	Major Observations	Reference
I. Natural	Chitosan	Hepa 1–6	Chitosan + PCL	Conventional electrospinning	Improved cell viability	[58]
		Primary rat hepatocytes	Surface modified with galactose	Conventional electrospinning	Improved functional activity of hepatocytes (increased Albumin, Urea secretion and improved P450 activity) on the galactosylated chitosan nanofibers	[61]
		Primary rat hepatocytes	-----	Conventional electrospinning	Albumin production increased 1.5 to 2 fold on the nanofiber scaffolds	[65]
		Primary rat hepatocytes	Fibronectin coating	Conventional electrospinning	CYP activity increased	[69]
	SILK	----	PEO + silk	Conventional electrospinning	Bead less Fiber formation of silk fibroin	[88]
II. Synthetic	PLA	HepG2	Lecithin incorporation	Conventional electrospinning	Increased cell proliferation	[67]
		----	PLA + PCL	Melt electrospinning	Fibers deposited onto pork liver for wound dressing applications	[45]
	PCL	HepG2	---	Conventional electrospinning	Comparison of cell viability on PCL mats and ECM tissue	[91]
		HepG2	Galactosylation and Chitosan incorporation	Conventional electrospinning	Improved cell growth and proliferation	[77]
		HepG2 And primary mouse hepatocytes	---	Conventional electrospinning	Increased Proliferation observed with changed fiber orientation whereas functions like albumin and CYP activity remained the same	[49]
		Human primary hepatocytes HUVECs	3D Printed and stacked	Melt electrospinning	Transplanted scaffolds improved survival and reversal of acute injury	[96]
		Primary rat hepatocytes	Gelatin and ECM incorporation	Conventional electrospinning	Increased albumin secretion	[95]
	PLLA	hMSCs	Gelatin incorporated	Conventional electrospinning	Increased albumin secretion and CYP3A4 activity	[81]
		HMSCs	Plasma treatment and collagen incorporation	Conventional electrospinning	Trans differentiation of MSCs into hepatocytes and increased albumin secretion up to 21 days	[82]
		Primary rat hepatocytes	NH3 Plasma treatment and Type I collagen incorporation	Conventional electrospinning	Hepatocyte aggregation observed, along with increased albumin urea secretion and CYP1A enzyme activity	[64]
		HepG2	Epithelial cell layer seeded and decellularized to obtain matrix incorporated scaffolds	Conventional electrospinning	Increased albumin, CYP and COLA1 gene expression on ECM decorated scaffolds	[93]
		THLE3	Decellularized human tissue ECM incorporated	Conventional electrospinning	Increased attachment and survival of cells, along with increased albumin secretion	[92]
	PLGA	Huh7.5	Collagen and fibronectin incorporated at different ratios	Wet electrospinning method	Viability, albumin secretion and CYP gene activity improved	[46]
		Primary human hepatocytes	Collagen and fibronectin incorporated at ratios mimicking matrix composition	Wet electrospinning method	3.5 fold increase in albumin gene expression and 4 fold increase in CYP Gene expression with the protein loaded scaffolds	[94]
	PCLEEP	Primary rat hepatocytes	Galactosylated surface	Conventional Electrospinning	Improved albumin secretion and P450 activity	[75]
		Primary rat hepatocytes	3-MC inducer of P450 loaded scaffolds	Conventional Electrospinning	1.5 fold increase in P450 activity	[80]
	PLACL	hMSCs	Collagen incorporation	Conventional Electrospinning	Increased expression of HNF4A and albumin	[87]

## Data Availability

Not applicable.
